# The Words of Affectivity. Affect, Category, and Social Evaluation Norms for 400 Polish Adjectives

**DOI:** 10.3389/fpsyg.2021.683012

**Published:** 2021-09-08

**Authors:** Szczepan J. Grzybowski, Miroslaw Wyczesany, Hanna Cichecka, Aleksandra Tokarska

**Affiliations:** ^1^Institute of Applied Psychology, Faculty of Management and Social Communication, Jagiellonian University, Kraków, Poland; ^2^Institute of Psychology, Faculty of Philosophy, Jagiellonian University, Kraków, Poland

**Keywords:** adjectives, affective norms, trait, state, word list, Polish language

## Abstract

Emotional adjectives can be grouped into two main categories: denoting and connoting stable (personality) traits and denoting and connoting transient (mood) states. They relate closely to the concept of affectivity, which is a pervasive tendency to experience moods of positive or negative valence. They constitute a rich study material for personality and affect psychology and neuroscience. Thus, this study was designed to establish a normed list of emotional adjectives with ratings encompassing four dimensions: emotional valence (positive or negative), emotional arousal (low-arousing or high-arousing), category (state, trait, and hybrid), and social judgment (competence, morality, and mixed). The adjectives were preselected based on previous broad Polish norming studies, personality and mood questionnaires, and a dictionary study. The results of the study were drawn from 195 participants who rated 400 adjectives that were chosen based on similar linguistic variables, such as frequency and word length. The dataset measures were proven to be stable and reliable. Correlations between the emotional valence and state-trait, valence and competence-morality, and emotional arousal and competence-morality dimensions were found. The study was successful in preparing a dataset of well-categorized (state, trait, and hybrid) positive and negative adjectives of moderate to high arousal ratings. Since the words were matched on linguistic variables, the set provided useful material that can be readily used for research into the effects of the category and emotional dimensions on language processing and as a basis for new personality questionnaires and mood checklists. The dataset could also be seen as a supplement for broader sets of published normed materials in Polish that link emotion and language.

## Introduction

### The Concept of Affectivity

The ability to categorize and communicate the emotions of oneself and others is of paramount importance to human beings in both personal and social contexts. The categorization, experience, and communication of these emotions are mostly based on lexical means, i.e., words. According to the lexical hypothesis, which is well-established in personality psychology, the most relevant and important aspects of human experiences (traits and differences) become part of a language, with the aspects of utmost importance and usefulness forming single words (Cattell, 1943). A better understanding of the linguistic means and ways (personal, social, and culturally specific) related to emotional experience has been one of the goals of psychologists, linguists, and cultural anthropologists for decades (Wierzbicka, 1994, 1999). A psychological concept that can help us navigate through the existing theoretical and empirical richness and complexities is affectivity, which is a somewhat forgotten framework proposed by Tellegen (1985) and Tellegen and Waller (2008) and then elaborated on by Watson (Watson and Clark, 1984; Watson and Tellegen, 1985). Essentially, affectivity is a stable tendency to experience particular mood states. It can be seen as a personality trait that closely relates to and depends on mood. The affective state, i.e., mood, can be structured by itself in a two-dimensional, orthogonal fashion as a positive and negative affect (Watson and Tellegen, 1985).

By encompassing two mood-dispositional dimensions, affectivity is then evidenced by a pervasive predisposition to experience either negative mood states, termed as negative affectivity (NA), or positive ones, termed as positive affectivity (PA). It is worth noting that both of these dimensions influence the broader aspects of our lives, such as self-concept and cognition (Watson et al., 1988). A person characterized by high NA tends to often experience sadness, tension, anxiety, worry, hostility, and disgust, whereas a high PA person is more likely to experience feelings of joy, enthusiasm, interest, mental alertness, and energy regularly. Negative affectivity correlates positively with depression and anxiety, whereas PA correlates negatively with depression only (Watson et al., 1988). High PA interestingly reflects a sense of well-being and competence, which then forms the important aspects of self-perception, self-worth, and engagement with others (Wojciszke, 1997).

Affectivity as a personal trait is based on and conveyed by emotional experiences perceived in the self and others as regularly occurring states. The regularity takes the form of a stable tendency to experience a broad set of states with similar emotional attributes or attributes that are described on similar dimensions. Two of the most important and rudimentary of these attributes, i.e., dimensions, are emotional valence (or pleasure–displeasure) and arousal (or activation–deactivation). In fact, these two attributes combined form a core effect, a basic, pre-conceptual form of human experience (Russell, 2003, 2009). It is worth noting that affectivity, in a broad scope, can be seen as the source of the observed tendency and the result of it. In other words, e.g., a highly neurotic person will often and be more prone to experiencing feelings of worry, tension, and anxiety because of personal constituents. Conversely, a person that often happens to experience such states due to circumstances, work environment, and other situations can become neurotic or be described as neurotic by others. Regardless of the cause-and-effect description, affectivity points to the importance and intricacy of emotional experience related to both traits and states. How people categorize, encode, comprehend, and convey such experiences seem to be as worthwhile and engaging as an endeavor now as it was decades ago. The most common and substantial means of “dealing” (in the abovementioned terms) with such experiences are verbal stimuli of one particular grammatical class, namely, adjectives.

### Adjectives as Means of Communicating Affect and Basis for Self-Others Evaluation

Aside from verbs and nouns, adjectives are the most important open-class words, which are present in most languages as they are used to describe persons, objects, situations, and phenomena denoted by nouns (Crystal, 2010). They also form the basis of the majority of personality questionnaires (e.g., the Big Five model itself; Goldberg, 1992; Costa and McCrae, 2008) and mood checklists, with either two-dimensional and emotional valence-based (with positive and negative affect; Watson and Tellegen, 1985), arousal-based (with energy and tension arousal; Thayer, 1989), or three-dimensional (linking the two mentioned, with energy, tension arousal, and hedonic tone; Matthews et al., 1990) being developed and used in psychology. In fact, most of the studies reporting norms for a large number of open-class words include adjectives (for an overview, see Table 1 in Riegel et al., 2015).

In Polish, as in other Indo-European languages (especially Germanic ones like English, German, and Dutch), the adjectives can be classified into five taxonomic groupings (Angleitner et al., 1990; Szarota, 1995): (1) dispositions or traits, e.g., “trustworthy,” (2) temporary conditions or states, including behavioral and bodily states such as “tired,” (3) social terms, i.e., roles and evaluations, e.g., “brotherly” and “unacceptable,” (4) physical characteristics and appearance, e.g., “tall,” and (5) terms of limited utility, i.e., technical or vague, e.g., “haphazard.” The first two groupings are closely connected to the concept of affectivity. Also of paramount importance for the present study is the fact that there is a substantial number of adjectives of a “hybrid” or ambiguous kind, i.e., having both a trait and a state reading (Angleitner et al., 1990). These adjectives can be called “state-like conditions” (Ortony et al., 1987) and present a frequent yet problematic subcategory of research material. Terms such as “active,” “happy,” “nervous,” “energetic,” and “optimistic” are common examples of “state-like conditions” words. Since they seem of particular interest to the topic of affectivity, these verbal stimuli are one of the focus points of the present study.

There have been few normative word battery studies that dealt with adjectives only (Gilet et al., 2012; Ric et al., 2013; Quadflieg et al., 2014). All of them involved French words and each focused on different aspects of verbal stimuli evaluation and categorization. The study of Gilet et al. (2012) presented data on age-related ratings on the valence, arousal, and imagery dimensions of trait and state adjectives, where no distinction between state and trait was made. In the study of Ric et al. (2013), they focused on trait adjectives alone and reported evaluations of valence, approach-avoidance, and possessor-other relevance dimensions. Finally, the study of Quadflieg et al. (2014) collected a broad set of adjectives, including trait, state, and appearance ones, alongside non-human descriptors and reported ratings on the human–non-human applicability, valence, visibility, intensity, familiarity, concreteness, and temporal stability dimensions. The temporal stability seemed to be a particularly interesting dimension, ranging from very transitory (state) to very enduring (attribute). Therefore, this study found that temporal stability increased alongside applicability to non-human entities; thus, it was a marker of object (inanimate) unchangeability rather than a human affective experience.

The two aforementioned groupings of adjective classification indeed seem the most pivotal for emotional experience and communication, both interpersonal and intrapersonal. However, the social evaluation, i.e., how one judges themselves and others in terms of ability and ethics, is of great importance as far as self-worth and engagement with others are concerned (Wojciszke, 2005), and can thus be related to strictly emotional measures. These social perceptions, i.e., evaluating oneself and others, are based on competence and morality judgments and are suggested to be the most important meanings processed by laymen within the contexts of social behavior, cognition, and personality (Wojciszke, 1997). These evaluations especially relate to trait words (Ric et al., 2013). In general terms, people tend to form self-judgments based more on competence values and other judgments based on morality values (Wojciszke, 1997, 2005; Wojciszke et al., 1998). Interestingly, moral transgressions are what always elicit negative emotions in the perceiver, whereas moral acts elicit positive responses, provided the person responsible for them is liked by the observer (Wojciszke and Szymków, 2003). Thus, social-based experiences and evaluations relate to affectivity, while the specifics of this relationship within the emotional language framework are well worth investigating. How the competence and morality evaluations within the social dimension relate to emotional experience conveyed by both trait and state aspects of affectivity is another point of interest of the present study.

### Affective Norms for Verbal Stimuli

To our best knowledge no previous normative word rating study combined category (trait, state), social (competence, morality) and emotional evaluations. Previous research presenting word batteries in Polish (Riegel et al., 2015; Imbir, 2016), upon which the present study is based, focused on important dimensions related to meaning and interplay between language and emotions with a broader and more general scope. The most common and well-researched factors of the affective kind, which are present in many research programs across cultures and languages, are valence (the pleasantness or unpleasantness of an object) and arousal (the internal reaction evoked by an object, ranging from calmness to extreme activation or excitement). It is worth noting that brain responses during emotional word processing have been shown to be modulated by both dimensions, either through independent or interactive manners (for a review see Citron, 2012). The origin of such an approach toward emotion-laden language could be traced to the semantic differential research by Osgood et al. (1957), in which variance in affective meaning assessments was mostly accounted for by three main dimensions: evaluation (rating something as good or bad and pleasant or unpleasant), i.e., valence, activity (active or passive and lively or still), which related closely to arousal, and potency or dominance (strong-controlling or weak-submissive).

One of the best known, and commonly used as the basis for research, normative databases is the Affective Norms for English Words (ANEW) collected by Bradley and Lang (1999), which has 1,034 words with norms for emotional valence, arousal, and dominance. Impressively, it has been extended by the study of Warriner et al. (2013) to 13,915 words. Another large affective word dataset, which includes 4,300 words, is available in Dutch (Moors et al., 2013). The Nencki Affective Word List (NAWL; Riegel et al., 2015), the Polish adaptation of the Berlin Affective Word List-Reloaded (BAWLR; Võ et al., 2009), is a list of 2,902 words rated on emotional valence, arousal, and imageability scales with control for linguistic variables (frequency, word length, and grammatical class). The study reported on 612 adjectives of various kinds, which is consistent with the groupings discussed above. The impressive Affective Norms for Polish Words Reload by Imbir (2016; ANPW_R), originally based on the ANEW list (Bradley and Lang, 1999), presented the assessments for 4,900 words on affective scales (valence, arousal, dominance, origin, and subjective significance) alongside psycholinguistic ones (concreteness, imageability, and age of acquisition) with linguistic variables control. There are 768 adjectives, again, of various groupings, which are reported there. This subset was the point of origin of the present study. In our approach, it was decided to abandon the psycholinguistic scales, i.e., the adjectives related to emotional experiences based on states and traits are rather more abstract and not readily imageable, and rather focus on the main affective ones (valence and arousal) and the essentials for the concept of affectivity categorical evaluations (state, trait, and hybrid).

### Aims

The main objective of the study was to provide research materials for studies on state- and trait-based affective experiences, which would help us to better understand the bottom-up (stimulus-based) and top-down (related to goals, attitudes, and experiences; Corbetta and Shulman, 2002) processes of emotion word encoding. In a broader theoretical framework, the present work was designed to provide us with a more complete description of affectivity and, as a result, enable us to better grasp the interplay between affective dispositions (personality traits) and states (moods) of varying valence and arousal levels. State-like conditions conveyed by “hybrid” adjectives should be of particular interest in this context for researchers, especially those dealing with very sensitive and finely detailed measures of stimuli encoding, e.g., evoked potentials of the brain (EP or event-related potential ERP). The state, trait, and hybrid adjective list could prove to be beneficial especially in the line of research that tries to distinguish and detail mood-congruence and disposition-congruence effects during stimuli processing and state-trait interactions (Rusting, 1998). Because of this, and in order to build a more reliable, stable, and ready to use research base, especially for sensitive brain responses (ERP) study programs, it was decided to select the adjectives on a stricter linguistic control basis (preselection, see section Materials and Methods). Such a list could also prove useful for developing new and updating old mood checklists and personality questionnaires.

The secondary goal of the study was to explore the relations between affective (and category) adjective norms and social judgment norms based on competence and morality evaluations. These evaluations form the basis for self- and other-perceptions and have probable, yet not clearly established, connections with the affective experience.

Similar to the case with other normative studies in Polish (Riegel et al., 2015; Imbir, 2016), it was predicted in this study that the dataset obtained would be reliable (split-half estimates) and stable (correlations with other databases, especially with the ratings of the ANPW_R, since the present study shared a bulk of adjectives with the set). The study also predicted that there will be a substantial number of adjectives categorized as hybrids, In an exploratory fashion, we also expected the social judgment ratings to correlate with other scales (possibly most notably with the affective ones, especially valence).

## Materials and Methods

### Participants

One hundred and ninety-five young adults who were students of Jagiellonian University with an *M* age of 22.36, *SD* = 2.23 participated in the study. Most of the participants were women (*N* = 150, *M* age 22.21, *SD* = 2.08), with a minority of men (*N* = 45, *M* age 22.87, *SD* = 2.63). The vast majority of participants were right-handed with only 10 participants declaring left-handedness (women *N* = 9, men *N* = 1). All of the participants signed a consent form before the procedure. After they completed the ratings, they were paid a Polish currency equivalent of *e*3.5 for their participation.

### Study Design and Materials

A special effort was made to prepare a more robust adjective list from those present in Polish word datasets (Riegel et al., 2015; Imbir, 2016), so that we could select the most representative and linguistically matched cases (preselection) from them. Firstly, all the adjectives from the larger ANPW_R (Imbir, 2016) list were extracted, then the list was supplemented with the adjectives from the NAWL (Riegel et al., 2015), which were not present on the ANPW_R. Afterward, two judges, one holding a Ph.D. in psychology and the second a Ph.D. in Polish literature, selected all of the adjectives denoting and connoting traits and states relating to human affective experience, in essence, personality, and mood adjectives from the compiled list. This yielded a list of 401 adjectives. Then, we extracted all the adjectives from the mood checklists (Watson and Tellegen, 1985; Thayer, 1989; Matthews et al., 1990) and the personality questionnaires and lexical studies on personality (Cattell, 1943; Goldberg, 1992; Costa and McCrae, 2008; see also De Raad, 2000) with translations based on the Polish adaptations of the tools and the individual differences manual of Strelau (Szarota, 1995; Strelau, 2014). Finally, the judges went through a small contemporaneous dictionary of the Polish language (Sobol, 2006) and selected from it all the mood and personality adjectives. The complete list comprised 1,061 adjectives. Linguistic variables for stricter control were obtained for all the adjectives: frequency values from two datasets, as was the case in the study of Imbir (2016), Subtlex_pl film and TV subtitle database (Mandera et al., 2015) and literature and electronic texts collection by Kazojć (2011) alongside word length values, i.e., letter count. Based on those values, 400 adjectives were then selected for normative ratings. The final list comprised the most average words, located effectively within 0.75 SD of the respective means^1^.

### Procedure

**Figure 1:**
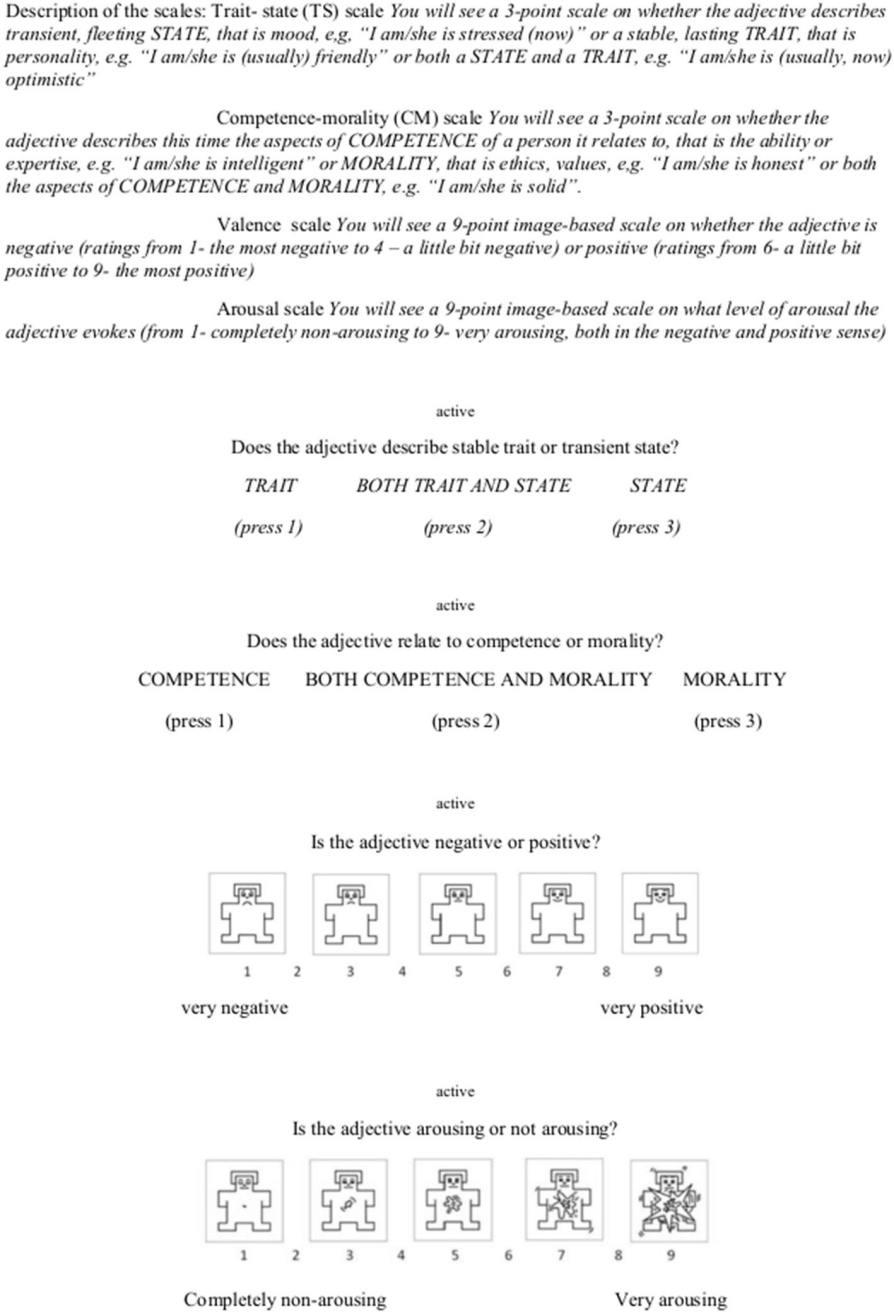
Descriptions of the scales seen during the introductory information period of the rating session and subsequent sample rating screens with each scale as presented to the participants. Self-Assessment Manikin (SAM) based on (Bradley and Lang, 1999).

The participants were divided into four groups, with each group required to rate 100 adjectives. The first group consisted of 48 participants (women *N* = 32), the second 51 (women *N* = 38), the third 48 (women *N* = 42), and the fourth 48 (women *N* = 39). All the groups rated the adjectives on the same four dimensions. Two of these dimensions were affective and exactly the same as seen in the study of Imbir (2016). Both were also based on the Self-Assessment Manikin (SAM) used in the ANEW ratings (Bradley and Lang, 1999) and used nine-point scales. The emotional valence dimension rating was between 1 (most negative) and 9 (most positive) and the emotional arousal was from 1 (completely non-arousing) to 9 (extremely arousing). The third dimension was a category one rated on a three-point scale, where 1 meant that the shown adjective denoted a stable trait, 3 a transient state, and 2 that it could denote both a trait and a state. The last dimension was a social judgment one, and it was constructed, respectively, in the same fashion as the category one, with 1 meaning that the shown adjectives denoted a competence evaluation, 3 a moral one, and 2 both competence and moral judgment. Figure 1 shows a sample word with the rating scales as seen by the participants during the rating session, alongside the description of the scales presented to the participants at the beginning.

After the participants arrived in the laboratory, they were seated comfortably in a separate room. Then, it was explained to them that they were to rate 100 words and were further asked to try to pay close attention to each word and not dwell too much on the answer. They were also warned that each rating was final and that it would not be possible to go back and answer again. The participants then signed the consent forms and the rating procedure was run in the PsychoPy2 software (University of Nottingham) (Peirce et al., 2019), where detailed instructions with the scales description were presented at the start. The participants read the instructions at their own pace and were encouraged to ask questions if anything was unclear. After the instructions were read, two sample words with full scales were presented; the samples were not a part of the 400 adjectives to be rated. Then, the actual rating session began. The participants rated each word on four scales by pressing the numerical keys on a keyboard. Each adjective was presented on the screen in a central position for 2 s, followed by the rating scales appearing, each one at a time, with the adjective to be rated visible in smaller fonts above the scale (see Figure 1). The next scale appeared immediately after the rating key was pressed. On average, it took 30 min to complete the 100-word rating. In the end, the participants were presented with a sheet of paper on which all 100 adjectives were printed and asked to mark the words that they did not comprehend. After that, the participants were thanked, paid, and were free to leave.

The procedure was prepared in two versions, namely, for women and men because Polish is a fusional language and has suffixes pointing to the gender of the nouns. Thus, an adjective “active,” “aktywny” in Polish, is a male form of the word (because of the suffix “y”), while the female form is “aktywna.” Afterward, all the 400 adjectives were presented in one form depending on the sex of the participant.

## Results

**Figure 2:**
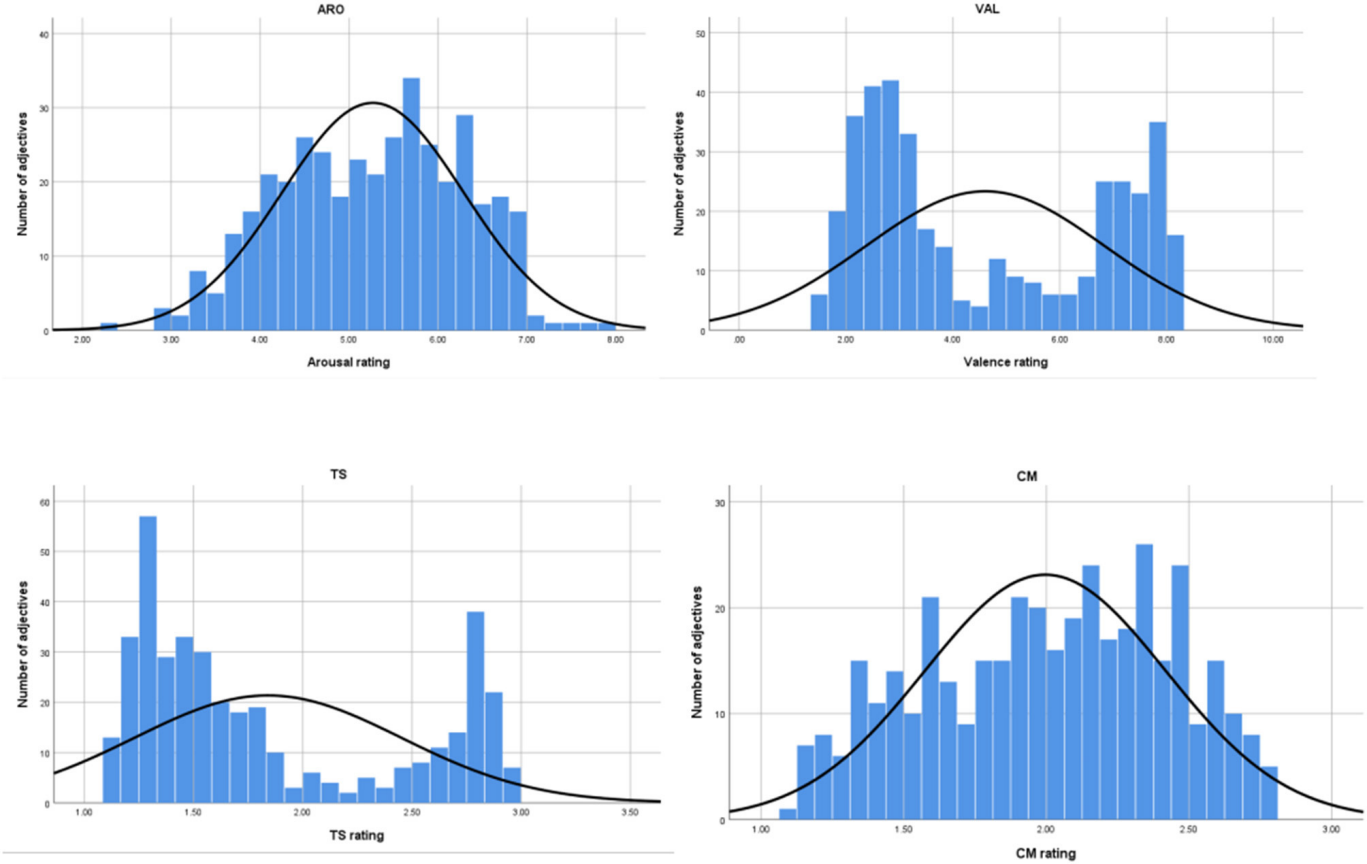
Histograms presenting the number of adjectives rated in one-point intervals of the scales of the respective dimensions' (ARO, arousal; VAL, valence; TS, trait-state; CM, competence-morality).

All statistical analyses were conducted in the IBM SPSS 26 software, New York, USA. In the beginning, descriptive statistics, i.e., *M* and *SD*s, for every adjective and rating scale [valence, arousal, trait-state (TS), and competence-morality (CM)] were obtained. The complete list of 400 adjectives (original and translated into English) with the statistics (alongside linguistic values for frequency and letter count) is available as Supplementary Material to the study. Afterward, the number of adjectives described as not understood at the end of the rating session was analyzed, and all the words marked as unintelligible by more than 25% of the participants who rated them were excluded from further analyses. The words that were not well-understood were: “afektowany” (“mincing”), “egzaltowany” (“exalted”), “lubieżny” (“lecherous”), “obcesowy” (“objectionable”), “sterany” (one of the synonymous variants of “weary”), “wystȩpny” (“debased”), “zapiekły” (“fiery”), and “zdrożony” (another of the synonymous variants of “weary”)^2^. Table 1 presents the general descriptions for the included 392 adjectives. In order to test for the normal distribution of the ratings, Shapiro–Wilk tests were used. All the rated dimensions were shown to have non-normal distributions. Figure 2 shows the distributions of the four dimensions ratings. Emotional valence and trait-state were bimodal (with TS being clearly negatively biased toward the lower end of the scale), whereas the distribution of CM and arousal only resembled normal distribution (with CM being centered close to the midline and arousal being slightly positively biased toward the high end of the scale).

**Table 1:** Descriptive statistics calculated for each dimension for all the intelligible words.

	***N***	**Min**	**Max**	**M**	**SD**
Arousal	392	2.40	7.86	5.2671	1.02080
Valence	392	1.40	8.33	4.6063	2.23256
Trait-state	392	1.10	2.98	1.8408	0.61003
Competence-morality	392	1.10	2.79	1.9961	0.42274

Each dimension was divided into low, moderate, and high or one category or mixed type based on the rating ranges of average scores. The emotional valence dimension was divided into low-valence (negatively rated) adjectives (ratings ranging from 1 to 3.99), moderate (neutral) adjectives (ratings from 4 to 6), and high-valence (positive) adjectives (ratings from 6.01 to 9). The criteria for this classification were based on previous studies on normed word datasets (Ferré et al., 2012; Monnier and Syssau, 2014; Imbir, 2016), which used the same SAM rating scales and divided the words similarly. Similarly, the likewise scaled emotional arousal dimension was divided into adjectives that were low (ratings from 1–3.99), moderate (4–6), and high (6.01–9) in arousal. The dimensions TS and CM were evaluated on simpler three-point scales, in which adjectives with the lowest ratings (from 1 to 1.49) were classified as trait and competence ones, adjectives with moderate ratings (from 1.5 to 2.5) were hybrid (TS) or mixed (CM), and the adjectives with the highest scores (1.51–3) were state and morality words, respectively. The justification for such ranges was that the single category evaluations, e.g., trait or state, are simple and obvious, meaning the categorical denotation of the word is easily grasped and so the population scores should be located close to the polar ends of the scales, i.e., low or high. In contrast, the hybrid and mixed category evaluations have a level of uncertainty *vis-à-vis* having a broader denotative and added connotative meaning, so their classification should be based on broader average scores across the tested population. The number of adjectives classified on the subdivided dimensions based on average rating score ranges is presented in Table 2.

**Table 2:** Numbers and percentages of the adjectives rated on every dimension (divided into low, moderate, and high or one category type or a mixed category based on the rating ranges).

	**Number of adjectives**	**% of the included adjectives (392)**
Arousal low Arousal moderate Arousal high	48 242 102	12 62 26
Valence low (negative) Valence moderate (neutral) Valence high (positive)	209 45 138	53.3 11.5 35.2
Trait Trait-state hybrid State	165 127 100	42 32.5 25.5
Competence Competence-morality mixed Morality	62 284 46	16 72 12

Reliability and stability analyses were conducted to assess the validity of the ratings. Finally, to verify the relations between the dimension correlation analyses were conducted, with an additional and tentative analysis of the differences between sexes at the end.

### Reliability

The reliability of the assessment was measured by applying the split-half estimation across the whole sample. The split was based on the odd and even numbering of the participants, with special care taken with respect to the less frequent men in order to balance their number in each half. The mean rating for each adjective in each group was then calculated and Pearson correlations were applied within each dimension. Because of the smaller number of participants in the split-half comparisons as compared to the whole set, the correlations were then adjusted through the use of the Spearman–Brown formula. All the correlations were strong (*r* > 0.9) and significant (*p* <0.01). Table 3 presents the results.

**Table 3:** Reliability and stability estimations for every dimension.

	**Split-half correlation (r-Pearson's)**	**Split-half correlation Spearman-Brown adjustment**	**Correlations with the ANPW_R**	**Correlations with the ANEW**	**Correlations with Quadflieg et al. (2014)**
Arousal	0.905	0.950	0.615	0.680	0.388
Valence	0.986	0.993	0.916	0.855	0.919
Trait-state	0.969	0.984	–	–	−0.760
Competence-morality	0.910	0.953	–	–	–

*All p <0.01*.

### Stability

To measure the stability of the ratings, *r*-Pearson correlations were applied for the 132 adjectives that were qualified for the final 400-word set and that were also a part of the ANPW_R dataset of Imbir (2016). Since only two scales (emotional valence and arousal) were exactly the same as what was used in the work of Imbir, those two affective dimensions were correlated. Both correlations were significant (*p* <0.01), but in particular, the emotional valence one was especially high (*r* = 0.916), whilst arousal was moderately strong (*r* = 0.615). To further validate the ratings, we additionally correlated word scales from our dataset with the adjectives from the English-based ANEW dataset (Bradley and Lang, 1999) and the biggest French-based adjective normed study (Quadflieg et al., 2014). This was achieved by matching the English translations of the adjectives. Correlations with the ANEW datasets, based on the 62 adjectives found in both datasets, were significant (*p* <0.01) and high; again, especially for the emotional valence dimension (*r* = 0.855) and the arousal dimension, but to a lesser extent (*r* = 0.68). The same was true for the correlations with the French study (all significance levels *p* <0.01), where, again, 132 matching adjectives were found, and we compared three dimensions: emotional valence (*r* = 0.919), emotional arousal, called “intensity” in the referenced dataset (*r* = 0.388), and temporal stability, related to the trait-state dimension of our study, only with reversed scaling (*r* = −0.760). All of the results related to the stability measures can also be seen in Table 3.

### Correlations Between Variables

In order to check for relations between the measures, *r*-Pearson correlations were applied for all the dimensions, i.e., affective, category, social judgment alongside linguistic variables). Because of the non-normal distribution of the variables, we did not conduct a partial correlation analysis. Table 4 presents all the results. Here only the most pertinent, significant, and strongest correlations are described.

**Table 4:** r-Pearson correlations between the variables.

	**Subtlex_pl frequency**	**Kazojć (2011) frequency**	**Number of letters**	**Valence**	**Arousal**	**TS**	**CM**
Subtlex_pl frequency	–	0.512^**^	−0.077	0.025	0.176^**^	−0.079	0.136^**^
Kazojć (2011) frequency	0.512^**^	–	−0.178^**^	0.118^*^	0.022	0.141^**^	0.056
Number of Letters	−0.077	−0.178^**^	–	−0.076	0.087	0.070	0.037
Valence	0.025	0.118^*^	−0.076	–	0.075	−0.230^**^	−0.305^**^
Arousal	0.176^**^	0.022	0.087	0.075	–	−0.076	0.250^**^
TS (trait-state)	−0.079	0.141^**^	0.070	−0.230^**^	−0.076	–	0.014
CM (competence-morality)	0.136^**^	0.056	0.037	−0.305^**^	0.250^**^	0.014	–

* *p = 0.05;*

** *p = 0.01*.

**Figure 3:**
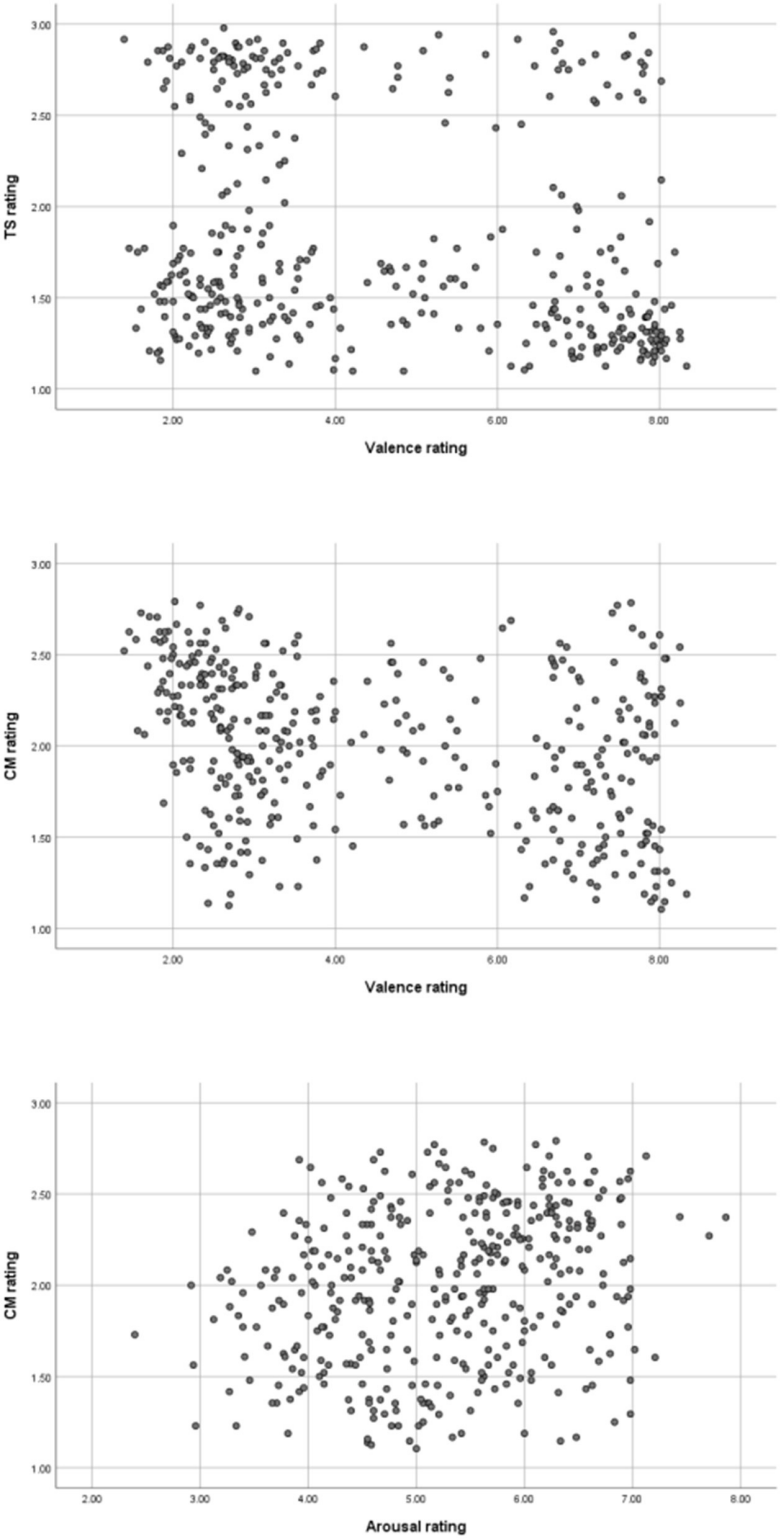
Scatterplots of the significant correlations between affective (valence and arousal), category (TS, trait-state), and social judgment (CM, competence-morality) dimension ratings.

The emotional valence dimension was found to be negatively correlated weakly and moderately with the TS (*r* = −0.23) and CM dimensions (*r* = −0.305), respectively, whilst arousal was weakly positively correlated with competence-morality (*r* = 0.25). Figure 3 shows the scatterplots of the correlated ratings. The emotional valence correlation with the category dimension would tentatively point to the fact that positive adjectives are categorized rather as trait ones, whereas valence relation to social judgement would mean that negative adjectives are judged as more related to morality. The correlation between emotional arousal and CM dimensions would, in turn, point to the observation that the more arousing adjectives are morality ones.

Additionally, comparisons between sexes were conducted (although one has to note the uneven number of male and female participants, so all the differences and relations reported here should be seen as tentative at best). The r-Pearson correlations of all the dimensions for all the included words as rated by female and male participants were applied. All of the rated dimensions were significantly and strongly positively correlated between the sexes: arousal *r* = 0.682 (*p* <0.01), valence *r* =0.958 (*p* <0.01), TS *r* = 0.905 (*p* <0.01), CM *r* = 0.789 (*p* <0.01). The possible differences between the ratings were verified by paired *t*-tests. However, only the arousal dimension ratings proved to be significant, *t*_(391)_ = 3.77 (*p* <0.001), with the adjectives being rated as more arousing by women (mean rating 5.29, SD = 1.29) than men (mean rating 5.11, SD = 1.14).

## Discussion

In a broader theoretical framework, the study was designed to describe in detail the linguistic means of encoding and communicating emotional experiences related closely to the mood-dispositional concept of affectivity (Watson and Clark, 1984; Tellegen, 1985). It can also be viewed as supplementary material to the two broad Polish word normative datasets, the ANPW_R (Imbir, 2016) and the NAWL (Riegel et al., 2015), focusing on one grammatical class of characteristics, which is particularly interesting for affective psychology and neuroscience, i.e., the adjectives denoting and connoting human traits and states. The dataset presented in the current study should be viewed as more closely related to the ANPW_R list since it shares 33% of the adjectives with it, two of the main affective scales (valence, arousal), and the linguistic measures (frequency, word length) used as the basis for the ratings.

Affectivity may be a very useful concept since it better orients and nuances our knowledge on the relationship between transient, state-like and stable, and trait-based emotional experiences. In terms of PA and NA, they also have applied values, whereas it is the former that can be successfully used as a diagnosis and prediction tool for depression (Watson et al., 1988) while both PA and NA can predict job performance (Kaplan et al., 2009). The analyses based on these observations could also prove useful for broader inferences on language and culture (Wierzbicka, 1994). In the case of the present study, the proportion analysis of the affective and category evaluations have shown that more than half of the selected 400 adjectives were of negative valence (53%) and nearly half (42%) denoted and connoted stable dispositions (traits). It is worth mentioning again that the 400 adjectives were the most average subset of a broad and representative sample of all the state and trait adjectives used in language and psychological research (see section Study Design and Materials). Future psycholinguistic and cross-cultural research could determine if such a pattern of relevance and importance embedded in the language (lexical hypothesis) focused more on the negative and fixed affective phenomena is indeed a real cultural characteristic, along with other patterns under what conditions that could possibly be observed.

In terms of a more tangible and immediate benefit, the dataset of the current study could prove useful for complex research into the mood or trait congruence aspects of emotional stimuli processing (Rusting, 1998). Since the adjectives were preselected on the most-representative basis of linguistic variables (double-sourced frequency values and word length values), the set could be especially well-suited for, e.g., pilot and main studies focused on sensitive stimuli processing measures, thus necessitating very strict matching and control of the independent variable, i.e., brain activity research programs, especially those using the evoked-potential technique.

In order to test the stability and reliability of the dataset, we used analyses similar to those reported in other Polish word norming studies (Riegel et al., 2015; Imbir, 2016). Although the present adjective-only study was much narrower in scope, it still required these kinds of analyses to properly validate it and establish its appropriateness and usefulness for further research. In fact, other adjective-based normed studies also measured the datasets for the reliability or both reliability and stability (Gilet et al., 2012; Quadflieg et al., 2014). We predicted that the measures would prove stable and reliable. This was indeed the case. The split-half assessment showed the dataset to be very reliable, as all the correlations noted in the analysis were very strong indeed. Based on the correlations with the same dimensions as seen in the ANPW_R, on the basis of a significant portion of the adjectives rated in the current study, the stability assessment proved to be high and satisfactory. However, it is worth noting that this was based only on two affective scales and not all of the 400 adjectives that were part of the estimation (in fact, it was 33% of the whole set). Also, the correlation score of the emotional arousal dimension was only moderately strong, which was probably due to the distribution of the arousal rating in the work of Imbir (2016), which had slightly different characteristics. Although also approximately normal in nature, it was clearly negatively biased (toward the less arousing ratings), whereas the distribution of arousal in the current study was slightly positively biased. Overall, the ANPW_R set consisted of less arousing stimuli. The same is true for the additional stability measures based on correlated data with English- and French-based datasets, which further validated our ratings (see quite similar correlations obtained with the ANEW to those with the ANPW_R, Table 3).

The French study (Quadflieg et al., 2014) comparison is especially interesting in this context. As in the case of the current research program, its database consisted of adjectives only and the number of matching adjectives between the two datasets was substantial (*n* = 132). The emotional valence correlation was high, as expected, whereas the arousal correlation, albeit still significant, was lower, mainly because of the lower average score (less arousing word set as a whole) and the dimension of intensity used in the French study carrying not quite the same denotations and connotations (how “extreme” and not “arousing” the meaning of each word was). Interestingly, we also correlated another similar dimension present in the dataset of the study of Quadflieg et al. (2014), namely, temporal stability, (mentioned in the introduction section) with the TS dimension of the current study, with the correlation proving to be high and significant. It was also negative, since stable and enduring dispositions (traits and attributes of the two scales, respectively) were scored on the low end of the TS and the high end of the temporal stability scale. This proves that the TS scale is an important and sensitive dimension of adjective-based semantics and that the transient vs. enduring aspect of concepts expressed through language is prominent and more general in nature since it is present in words relating to human experiences and dispositions and in those relating to the attributes of inanimate objects.

As a side note, most studies cross-validating their ratings with other datasets have found that emotional valence correlations are stronger than arousal correlations (Warriner et al., 2013; Riegel et al., 2015; Imbir, 2016). This was clearly the case in the present study (see Table 3). Emotional valence seems to generalize very well across languages and research programs, whilst arousal shows more variability, possibly due to its stronger dependence on situational aspects, the broader connotations it evokes (which may provoke “the lost in translation” effects across languages), and its relation to affective states (the intensity of the actual feelings during the rating sessions).

This study also predicted that the category evaluation would reveal a substantial number of hybrid adjectives, i.e., denoting and connoting both trait- and state-based experiences, depending on the reading, which could be of high practical value in the language; further providing possible confounds in research (see above Rusting, 1998). Indeed, 32.5% of all the (comprehensible) adjectives rated were of this kind. This means that the hybrid adjectives were the second most abundant type of words related to affectivity (trait ones being the most common). Since our research material was sourced in various ways, one of which was a dictionary study, the proportion observed here seems like a good representation of the contemporary Polish language content. Because of this, and making use of the two original evaluations (category and social judgment), the current study is differentiated from other adjective-only based normed datasets (Gilet et al., 2012; Ric et al., 2013; Quadflieg et al., 2014) and, based on the category evaluation, it can be of applied value as a source of essential material for new or updated personality questionnaires and mood checklists.

As is the case with all the word normative research, we analyzed the relations between the measures used in the study. First of all, we did not find a significant correlation between the affective dimensions (valence and arousal), which is surprising, since it is usually strongly evidenced in most of the previous word rating research (Gilet et al., 2012; Quadflieg et al., 2014; Riegel et al., 2015; Imbir, 2016). This correlation typically points to a U-shaped relationship between emotional valence and arousal (Bradley and Lang, 1999; Moors et al., 2013; Warriner et al., 2013), meaning the more extreme the valence rating (either positive or negative), the higher the arousal score of a given stimulus. The words neutral in their emotional valence are typically rated moderately or low on their arousal. However, the lack of correlation in the present study can be explained by the nature of the preselected word set. We based the aims of the study and our search for the materials on the affectivity concept, which, at its core, has two orthogonal dimensions of positive and negative affect (Watson and Tellegen, 1985). Those dimensions are varied in arousal within their poles. Therefore, both high scores on the valence scale (positive evaluations) and low scores (negative evaluations) dominating in our dataset (there are few neutral words relating to personality and mood in the language) relate to words of moderate to strong arousal ratings (see the numbers of arousal-rated adjectives in Table 2 and the distributions in Figure 2). In other words, the emotional valence dimension is clearly bimodal, with very few neutral cases, whilst the arousal dimension approximates normal distribution, with a mild positive bias. In such a clear-cut manner, this was not the case in other normative studies for vast arrays of words of different grammatical classes, including the ANPW_R (Imbir, 2016).

This study found weak to moderate correlations between the category and emotional valence dimensions and between the social judgment and valence dimensions, the latter of which was predicted based on research in social and personality psychology (Wojciszke et al., 1998; Wojciszke and Szymków, 2003). It was also the strongest observed (pertinent) correlation between measures (albeit still moderate at best, see Table 4)^3^. In essence, it points to a relationship in which the negative adjectives are rated as rather morality-based (see scatterplots, Figure 3). This is very interesting in the context of self–other perception and self–other evaluation (impressions and emotions), in which one tends to judge oneself on the competence basis and others on the morality basis (Wojciszke, 2005). If the negative adjectives embedded in language tend to be more related to morality aspects of social judgments, then it could follow that one has a (bottom-up and lexicon-based) bias toward evaluating others in a negative light. If the converse is true, then we speak mostly positively about ourselves, which could be one of the reasons behind a known characteristic of healthy people, namely, the fact that they declare feeling good and happy about themselves and their lives (Diener and Diener, 1996). These fascinating interconnected aspects of language, emotion, and social evaluations certainly merit further research. However, one of the limitations of the study must be mentioned. The three-point scale of the competence-morality dimension may be the source of some confusion and probably “flattened” the richness of this aspect. The scale was conceived as a counterpart to the category three-point scale, which seems to be a good enough measure for the purposes of the current study (see the numbers of the category ratings in Table 2). We wanted to avoid adding another, differently balanced scale, e.g., a four-point scale with an additional position, “neither” competence nor morality, to not tire and unnecessarily confuse the participants with different numbering (key press) systems. This was an oversimplification that might have worked disadvantageously for the study, thus, affecting the results, since the vast majority of the adjectives rated on the scale were evaluated as both competence and morality ones. Some of the participants reported at the end of the rating session that they picked this middle response because there was no response labeled “neither.” Such an option would be beneficial for any rating scale based on intricate evaluations, and social judgement is no exception. On the other hand, “neither” or “do not know” answers present in the scale could result in an “easy way out” for the tired participants rating dozens of words, which could further distort the evaluations. Therefore, 5-, 7-, or even 9-point scales seem like better solutions for a nuanced and complex dimension such as social judgments. This limitation must be strongly considered when interpreting all the aspects related to the CM measure.

Additionally, we looked for tentative relations in ratings between participants who were women and men, since the word norms for both sexes, although correlated, tended to differ especially *vis-à-vis* the affective variables (Riegel et al., 2015; Imbir, 2016). This study applied only the simplest of solutions and, thus, urge caution in interpreting the data since the groups of women and men were uneven in number. Sex-based ratings on all the dimensions were similar and highly positively correlated. Only the emotional arousal correlation was of moderate strength. By comparing the means of the ratings, we found that, indeed, the arousal rating differed significantly between the sexes, with higher ratings on average for all the adjectives evaluated by participants who were women. It could be a slight encouragement for further verification of the possibility that women are more sensitive to the emotional (arousing) content of words and, thus, evaluated them differently based on that variable. However, the picture is certainly more complex and possibly based on the interactive effects of at least valence and arousal in between sex comparisons (Riegel et al., 2015). The uneven woman–man number of participants is another limitation of the study. Although the ratings were similar and positively correlated to a high degree (and so the male ratings were included in the general dataset), the list should be viewed as more representative for the women population.

Lastly, the overall number of the adjectives included in the database (*n* = 400) may seem small, especially when one considers using words of this grammatical class in complex research programs, which require matching the words on several psycholinguistic conditions or filling the given sub-category with several dozens of stimuli for comparisons. Four hundred may be too small a number to begin with when considering the satisfaction of all the conditions. On the other hand, one of the presumed strengths of this dataset is its compactness and “pre-matching” of the linguistic variables that influence greatly early stages of stimuli encoding, namely, word length and frequency, which should make devising research programs easier.

## Data Availability Statement

The original contributions presented in the study are included in the article/Supplementary Material, further inquiries can be directed to the corresponding author/s.

## Ethics Statement

Ethical review and approval was not required for the study on human participants in accordance with the local legislation and institutional requirements. The patients/participants provided their written informed consent to participate in this study.

## Author Contributions

SG contributed to the study design, the analysis and interpretation of data, and the drafting and critical revision of the manuscript. MW contributed to the study design and the gathering of the data. HC contributed to the gathering of the data and the critical revision of the manuscript. AT contributed to the gathering of the data. All authors contributed to the article and approved the submitted version.

## Funding

This work was supported by a grant from the National Science Centre, Poland, 2016/23/D/HS6/01645.

## Conflict of Interest

The authors declare that the research was conducted in the absence of any commercial or financial relationships that could be construed as a potential conflict of interest.

## Publisher's Note

All claims expressed in this article are solely those of the authors and do not necessarily represent those of their affiliated organizations, or those of the publisher, the editors and the reviewers. Any product that may be evaluated in this article, or claim that may be made by its manufacturer, is not guaranteed or endorsed by the publisher.

## Footnotes

^1^The frequency values of the adjectives qualified for the ratings were between 1 and 669 in Subtlex_pl dataset and 1 and 768 in the collection of Kazojć. For a word to be excluded from the final set, it had to have a frequency below 1 and above 1,100 on just one dataset or exceed the upper frequency value on both datasets. The word length of the adjectives qualified was between 7 and 11 letters.

^2^Interestingly, but not surprisingly, the absolutely least understood word was “mincing,” which was described as incomprehensible by 81% of the participants rating the word.

^3^The strongest correlation was the obvious one, of interest only strictly linguistically, between two frequency measure subsets.

## Supplementary Material

Supplementary Table 1 List of 400 adjectives and their ratings on valence, arousal, category, and social judgments dimensions alongside linguistic (frequency and word length) measures.

## References

[B1] Angleitner A. Ostendorf F. John O. P. (1990). Towards a taxonomy of personality descriptors in German: a psycho-lexical study. Eur. J. Pers. 4, 89–118.

[B2] Bradley M. M. Lang P. J. (1999). Affective Norms for English words (ANEW): Stimuli, Instruction Manual, and Affective Ratings. Technical report C-1, The Center for Research in Psychophysiology, University of Florida, Gainesville, FL.

[B3] Cattell R. B. (1943). The description of personality: basic traits resolved into clusters. J. Abnormal Soc. Psychol. 38, 476–506. 10.1037/h0054116

[B4] Citron F. M. M. (2012). Neural correlates of written emotion word processing: a review of recent electrophysiological and hemodynamic neuroimaging studies. Brain Lang. 122, 211–226. 10.1016/j.bandl.2011.12.007 22277309

[B5] Corbetta M. Shulman G. L. (2002). Control of goal-directed and stimulus-driven attention in the brain. Nat. Rev. Neurosci. 3, 201–215. 10.1038/nrn755 11994752

[B6] Costa P. T. Jr. McCrae R. R. (2008). The revised NEO personality inventory (NEO-PI-R), in The SAGE Handbook of Personality Theory and Assessment, Vol 2: Personality Measurement and Testing, eds Boyle G. J. Matthews G. Saklofske D. H. (London: Sage Publications, Inc.), 179–198. 10.4135/9781849200479.n9

[B7] Crystal D. (2010). The Cambridge Encyclopedia of Language. Cambridge: Cambridge University Press.

[B8] De Raad B. (2000). The Big Five Personality Factors: The Psycholexical Approach to Personality. Gottingen: Hogrefe and Huber Publishers.

[B9] Diener E. Diener C. (1996). Most people are happy. Psychol. Sci. 7, 181–185. 10.1111/j.1467-9280.1996.tb00354.x

[B10] Ferré P. Guasch M. Moldovan C. Sánchez-Casas R. (2012). Affective norms for 380 Spanish words belonging to three different semantic categories. Behav. Res. Methods 44, 395–403. 10.3758/s13428-011-0165-x 22042646

[B11] Gilet A.-L. Grühn D. Studer J. Labouvie-Vief G. (2012). Valence, arousal, and imagery ratings for 835 French attributes by young, middle-aged, and older adults: the French Emotional Evaluation List (FEEL). Eur. Rev. Appl. Psychol. 62, 173–181. 10.1016/j.erap.2012.03.003

[B12] Goldberg L. R. (1992). The development of markers for the Big-Five factor structure. Psychol. Assess. 4, 26–42. 10.1037/1040-3590.4.1.26

[B13] Imbir K. K. (2016). Affective norms for 4900 Polish words reload (ANPW_R): assessments for valence, arousal, dominance, origin, significance, concreteness, imageability and, age of acquisition. Front. Psychol. 7:1081. 10.3389/fpsyg.2016.01081 27486423 PMC4947584

[B14] Kaplan S. Bradley J. C. Luchman J. N. Haynes D. (2009). On the role of positive and negative affectivity in job performance: a meta-analytic investigation. J. Appl. Psychol. 94, 162–176. 10.1037/a0013115 19186902

[B15] Kazojć J. (2011). Słownik Frekwencyjny Jezyka Polskiego [Polish Language Frequency Dictionary]. Available online at: https://doczz.pl/doc/911406/słownik-frekwencyjny-jezyka-polskiego-v.05.2011

[B16] Mandera P. Keuleers E. Wodniecka Z. Brysbaert M. (2015). Subtlex-pl: subtitle-based word frequency estimates for Polish. Behav. Res. Methods 47, 471–483. 10.3758/s13428-014-0489-4 24942246

[B17] Matthews G. Jones D. M. Chamberlain A. G. (1990). Refining the measurement of mood: the UWIST Mood Adjective Checklist. Brit. J. Psychol. 81, 17–42. 10.1111/j.2044-8295.1990.tb02343.x

[B18] Monnier C. Syssau A. (2014). Affective norms for french words (FAN). Behav. Res. Methods 46, 1128–1137. 10.3758/s13428-013-0431-1 24366716

[B19] Moors A. De Houwer J. Hermans D. Wanmaker S. van Schie K. Van Harmelen A.-L.. (2013). Norms of valence, arousal, dominance, and age of acquisition for 4,300 Dutch words. Behav. Res. Methods 45, 169–177. 10.3758/s13428-012-0243-8 22956359

[B20] Ortony A. Clore G. L. Foss M. A. (1987). The referential structure of the affective lexicon. Cogn. Sci. 11, 341–364. 10.1016/S0364-0213(87)80010-1

[B21] Osgood C. E. Suci G. J. Tannenbaum P. (1957). The Measurement of Meaning. (Chicago, IL: University of Illinois Press).

[B22] Peirce J. Gray J. R. Simpson S. MacAskill M. Höchenberger R. Sogo H.. (2019). PsychoPy2: experiments in behavior made easy. Behav. Res. Methods 51, 195–203. 10.3758/s13428-018-01193-y 30734206 PMC6420413

[B23] Quadflieg S. Michel C. Bukowski H. Samson D. (2014). A database of psycholinguistic and lexical properties for French adjectives referring to human and/or nonhuman attributes. Can. J. Exp. Psychol. 68, 67–76. 10.1037/cep0000001 24001094

[B24] Ric F. Alexopoulos T. Muller D. Aubé B. (2013). Emotional norms for 524 French personality trait words. Behav. Res. Methods 45, 414–421. 10.3758/s13428-012-0276-z 23263927

[B25] Riegel M. Wierzba M. Wypych M. Zurawski Ł. Jednoróg K. Grabowska A.. (2015). Nencki affective word list (NAWL): the cultural adaptation of the Berlin Affective Word List–Reloaded (BAWL-R) for Polish. Behav. Res. Methods 47, 1222–1236. 10.3758/s13428-014-0552-1 25588892 PMC4636524

[B26] Russell J. A. (2003). Core affect and the psychological construction of emotion. Psychol. Rev. 110, 145–172. 10.1037/0033-295x.110.1.145 12529060

[B27] Russell J. A. (2009). Emotion, core affect, and psychological construction. Cogn. Emot. 23, 1259–1283. 10.1080/0269993090280937512529060

[B28] Rusting C. L. (1998). Personality, mood, and cognitive processing of emotional information: Three conceptual frameworks. Psychol. Bull. 124, 165–196. 10.1037/0033-2909.124.2.165 9747185

[B29] Sobol E. (2006). Słownik Jezyka Polskiego PWN [Polish Dictionary]. Wydawnictwo Naukowe PWN.

[B30] Strelau J. (2014). Róznice Indywidualne. Historia – Determinanty – Zastosowania. Scholar. Available online at: https://scholar.com.pl/en/books/1528-roznice-indywidualne-historia—determinanty—zastosowania-wydanie-drugie.html

[B31] Szarota P. (1995). Polska Lista Przymiotnikowa (PLP): Narzedzie do diagnozy Pieciu Wielkich czynników osobowości. [Polish Adjective List: an instrument to assess the five-factor model of personality.]. Studia Psychol. 33, 227–256.

[B32] Tellegen A. (1985). Structures of mood and personality and their relevance to assessing anxiety, with an emphasis on self-report, in Anxiety and the Anxiety Disorders, eds Tuma A. H. Maser J. (New York, NY: Lawrence Erlbaum Associates, Inc.), 681–706.

[B33] Tellegen A. Waller N. G. (2008). Exploring personality through test construction: development of the multidimensional personality questionnaire, in The SAGE Handbook of Personality Theory and Assessment, Vol 2: Personality Measurement and Testing, eds Boyle G. J. Matthews G. Saklofske D. H. (London: Sage Publications, Inc.), 261–292. 10.4135/9781849200479.n13

[B34] Thayer R. E. (1989). The Biopsychology of Mood and Arousal. (Oxford: Oxford University Press).

[B35] Võ M. L. H. Conrad M. Kuchinke L. Urton K. Hofmann M. J. Jacobs A. M. (2009). The Berlin affective word list reloaded (BAWL-R). Behav. Res. Methods 41, 534–538. 10.3758/BRM.41.2.534 19363195

[B36] Warriner A. B. Kuperman V. Brysbaert M. (2013). Norms of valence, arousal, and dominance for 13,915 English lemmas. Behav. Res. Methods 45, 1191–1207. 10.3758/s13428-012-0314-x 23404613

[B37] Watson D. Clark L. A. (1984). Negative affectivity: the disposition to experience aversive emotional states. Psychol. Bull. 96, 465–490. 10.1037/0033-2909.96.3.465 6393179

[B38] Watson D. Clark L. A. Carey G. (1988). Positive and negative affectivity and their relation to anxiety and depressive disorders. J. Abnormal Psychol. 97, 346–353. 10.1037/0021-843X.97.3.346 3192830

[B39] Watson D. Tellegen A. (1985). Toward a consensual structure of mood. Psychol. Bull. 98, 219–235. 10.1037/0033-2909.98.2.219 3901060

[B40] Wierzbicka A. (1994). Emotion, language, and cultural scripts, in Emotion and Culture: Empirical Studies of Mutual Influence, eds Kitayama S. Markus H. R. (American Psychological Association), 133–196.

[B41] Wierzbicka A. (1999). Emotions Across Languages and Cultures: Diversity and Universals. Cambridge: Cambridge University Press.

[B42] Wojciszke B. (1997). Parallels between competence- versus morality-related traits and individualistic versus collectivistic values. Eur. J. Soc. Psychol. 27, 245–256. 10.1002/(SICI)1099-0992(199705)27:3<245::AID-EJSP819>3.0.CO;2-H

[B43] Wojciszke B. (2005). Morality and competence in person- and self-perception. Eur. Rev. Soc. Psychol. 16, 155–188. 10.1080/10463280500229619

[B44] Wojciszke B. Dowhyluk M. Jaworski M. (1998). Moral competence-related traits: how do they differ? Polish Psychol. Bull. 29, 283–294.

[B45] Wojciszke B. Szymków A. (2003). Emotions related to others' competence and morality. Polish Psychol. Bull. 34, 135–142.

